# Gray’s Anatomy

**Published:** 1906-02

**Authors:** 


### BOOK REVIEWS.

GRAY'S ANATOMY—DESCRIPTIVE AND SURGICAL. A Text-Book of Human Anatomy. By Henry Gray, F.R.S., Lecturer on Anatomy at St. George's Hospital, London. New American from the fifteenth English Edition. Thoroughly revised and largely rewritten by J. Chalmers Da Costa, M.D., Professor of Surgery in Jefferson Medical College, Philadelphia, with the collaboration of a corps of specially trained assistants. Sixteen hundred pages, with eleven hundred and thirty-two illustrations in black and colors. Price, with illustrations in black and colors, cloth, $6.00, net; full leather, $7.00, net. Price, with illustrations in black, cloth, $5.50, net; full leather, 6.50, net. Lea Brothers & Co., Publishers, Philadelphia, New York.

Anatomy may well be considered the "gate to the practice of medicine." Those who pass successful examinations in it usually continue their medical course while those who fail frequently abandon medicine as a study forever. In passing this college crisis no book in any language has been of greater assistance to the student, and later to the practitioner, than the well-known work of Henry Gray.

Although dying young, he left a masterpiece which showed not only his profound knowledge of the human structure, but also his wonderful skill in presenting this knowledge for ready assimilation by other minds.

The original purpose of the author has been adhered to by Dr. Da Costa, who was eminently fitted for the task of revision for this New American Edition, which contains all the recent advances in anatomical knowledge.

The colored engravings are in keeping with the general excellence of the work, and of the eleven hundred and thirty-two illustrations no fewer than five hundred are new.

The special articles on Histology and Embryology have been wisely eliminated from this edition, as no single volume can offer adequate instruction on these subjects and Anatomy.

The new nomenclature has been introduced in parentheses, fol lowing the names still in current use in English-speaking countries.

